# The Tudor-domain protein TDRD7, mutated in congenital cataract, controls the heat shock protein HSPB1 (HSP27) and lens fiber cell morphology

**DOI:** 10.1093/hmg/ddaa096

**Published:** 2020-05-18

**Authors:** Carrie E Barnum, Salma Al Saai, Shaili D Patel, Catherine Cheng, Deepti Anand, Xiaolu Xu, Soma Dash, Archana D Siddam, Lisa Glazewski, Emily Paglione, Shawn W Polson, Shinichiro Chuma, Robert W Mason, Shuo Wei, Mona Batish, Velia M Fowler, Salil A Lachke

**Affiliations:** 1 Department of Biological Sciences, University of Delaware, Newark, DE 19716, USA; 2 School of Optometry, Indiana University, Bloomington, IN 47405, USA; 3 Nemours Biomedical Research Department, Alfred I duPont Hospital for Children, Wilmington, DE 19803, USA; 4 Center for Bioinformatics & Computational Biology, University of Delaware, Newark, DE 19716, USA; 5 Institute for Frontier Medical Sciences, Kyoto University, Kyoto 606-8507, Japan; 6 Department of Medical and Molecular Sciences, University of Delaware, Newark, DE 19716, USA

## Abstract

Mutations of the RNA granule component TDRD7 (OMIM: 611258) cause pediatric cataract. We applied an integrated approach to uncover the molecular pathology of cataract in *Tdrd7−/−* mice. Early postnatal *Tdrd7−/−* animals precipitously develop cataract suggesting a global-level breakdown/misregulation of key cellular processes. High-throughput RNA sequencing integrated with iSyTE-bioinformatics analysis identified the molecular chaperone and cytoskeletal modulator, HSPB1, among high-priority downregulated candidates in *Tdrd7−/−* lens. A protein fluorescence two-dimensional difference in-gel electrophoresis (2D-DIGE)-coupled mass spectrometry screen also identified HSPB1 downregulation, offering independent support for its importance to *Tdrd7−/−* cataractogenesis. Lens fiber cells normally undergo nuclear degradation for transparency, posing a challenge: how is their cell morphology, also critical for transparency, controlled post-nuclear degradation? HSPB1 functions in cytoskeletal maintenance, and its reduction in *Tdrd7−/−* lens precedes cataract, suggesting cytoskeletal defects may contribute to *Tdrd7−/−* cataract. In agreement, scanning electron microscopy (SEM) revealed abnormal fiber cell morphology in *Tdrd7−/−* lenses. Further, abnormal phalloidin and wheat germ agglutinin (WGA) staining of *Tdrd7−/−* fiber cells, particularly those exhibiting nuclear degradation, reveals distinct regulatory mechanisms control F-actin cytoskeletal and/or membrane maintenance in post-organelle degradation maturation stage fiber cells. Indeed, RNA immunoprecipitation identified *Hspb1* mRNA in wild-type lens lysate TDRD7-pulldowns, and single-molecule RNA imaging showed co-localization of TDRD7 protein with cytoplasmic *Hspb1* mRNA in differentiating fiber cells, suggesting that TDRD7–ribonucleoprotein complexes may be involved in optimal buildup of key factors. Finally, *Hspb1* knockdown in *Xenopus* causes eye/lens defects. Together, these data uncover TDRD7’s novel upstream role in elevation of stress-responsive chaperones for cytoskeletal maintenance in post-nuclear degradation lens fiber cells, perturbation of which causes early-onset cataracts.

## Introduction

Cataract, the loss of transparency of the eye lens, is the leading cause of blindness worldwide (1, 2). Depending on age of onset, cataract is classified as pediatric- or age-related. Although the former are far less common than the latter, occurring in frequencies of ~1–6/10 000 live births, they account for approximately one-tenth of childhood blindness worldwide (3–7). If untreated, congenital cataract can lead to sensory deprivation amblyopia and, if treated by surgery, can lead to complications throughout life (8, 9). It is estimated that between 8.3 and 25% of congenital cataracts are inherited (10) and can present as the primary phenotype in isolated or non-syndromic cases or as one of the multiple phenotypes in syndromic cases (11). Isolated inherited cataracts have been mapped to 48 genetic loci, and causative mutations in 36 genes have been identified (2, 12). Although age-related cataract (ARC) is multifactorial, understanding the genetic basis and molecular pathogenesis of congenital cataracts may offer new insights into the factors with implications for ARC (2). Toward these goals, animal models such as mice carrying specific targeted gene deletions have been utilized for elucidating the molecular function of these candidate genes in the lens, thereby informing on the distinct pathological mechanisms of cataract (12–16).

We recently reported that mutations in the ribonucleoprotein/RNA granule component gene *TDRD7* (OMIM: 611258) cause congenital cataracts, suggesting that regulators of post-transcriptional gene expression control have key function in vertebrate lens development and maintenance of transparency (17–21). Subsequent to our report, new human mutations in *TDRD7* have been linked to cataract in independent studies (22, 23). Moreover, a *TDRD7* polymorphism has been associated with age-related cataracts in a Han Chinese population (24), and expression profiling analyses have demonstrated a significant downregulation of *TDRD7* transcripts in human aged cataractous lenses (25), providing further evidence for the importance of this gene in lens development and homeostasis.

TDRD7 contains three Oskar-Tdrd7-Helix-Turn-Helix (OST-HTH)/Limkain, Oskar and Tudor containing proteins 5 and 7 (LOTUS) domains and three Tudor domains. The Tudor domains are predicted to bind to methylated arginine residues within other proteins (26). While the OST-HTH/LOTUS domains are predicted to bind to RNA (27, 28), recent reports indicate that they interact with DEAD-box helicases (29, 30), which function in regulating multiple aspects of RNA metabolic processes (31). Indeed, TDRD7 has been shown to be a component of ribonucleoprotein complexes and RNA granules in differentiating lens and sperm cells, and interestingly, *Tdrd7* deficiency in mice causes two prominent fully penetrant defects involving both of these distinct cell types, namely, cataracts and male-specific sterility resulting from azoospermia (17, 23, 32). These data suggest that TDRD7 has a critical role in lens development.

Characterization of the cataract phenotype in *Tdrd7*-deficient mice is expected to provide significant insights into the pathophysiology of this human developmental defect (17, 23, 32). In previous studies, HSPB1, SPARC, EPHA2 and CRYBB3 were found to be reduced in the *Tdrd7* null lens based on microarray data. However, this analysis did not involve any proteome or RNA-Seq analyses, and microarrays involve inherent limitations (probe-binding kinetics, dependency on known genes, etc.). Therefore, in the present study, we sought to address these technical limitations of the time. Here, we apply a multidisciplinary approach to elucidate the molecular and cellular basis of lens pathology exhibited in germline *Tdrd7*-targeted knockout mice (*Tdrd7^tm1.1Chum^*, hereafter referred as *Tdrd7−/−*). We carefully characterized *Tdrd7−/−* lens defects by histology, scanning electron microscopy and grid imaging, so we could temporally identify the onset of cataract to minimize the identification of indirect/secondary targets. Furthermore, we took unbiased omics-level approaches on both RNA and protein levels and applied RNA-Seq and 2D-DIGE analysis to examine the *Tdrd7−/−* lens.

The majority of the normal lens tissue is made of terminally differentiated fiber cells, which are derived from cells in the anterior lens epithelium (16). During their differentiation, fiber cells undergo degradation of nuclei and other organelles, which is necessary for minimizing light scatter for the development of lens transparency (33, 34). Our new data provide a molecular explanation to address long-standing questions on the mechanism of how fiber cells undergoing organelle degradation are able to prevent degradation of specific factors such as those associated with the cytoskeleton, thereby allowing the maintenance of normal fiber cellular morphology, especially in late maturation stages of differentiation (33). Our data identify a novel function for TDRD7 in the regulation of fiber cell morphology, specifically in cells post-nuclear degradation. In particular, these results provide additional robust support for *Hspb1* (*Hsp27*) mRNA to be downstream of TDRD7 protein in the lens. The previous work shows that HSPB1 is one of the constitutively highly expressed small heat shock proteins in humans that is involved in the regulation of cytoskeleton under stress conditions, among other cellular processes, and is linked to various pathological conditions (35). However, its significance to eye and lens development was not examined. Here, we show for the first time that HSPB1 knockdown causes microphthalmia and lens defects in a *Xenopus* model, and these defects can be rescued by mouse HSPB1 ectopic expression. These findings offer evidence that HSPB1 functions in eye and lens development. Together, these new findings represent a significant advance as they suggest a Tudor-family protein is upstream of HSPB1 expression. We propose a model wherein fiber cells that have undergone organelle degradation—and can be considered to face cellular challenges resembling a stress-like condition—need TDRD7 to sustain optimally high levels of the chaperone protein HSPB1 (HSP27), which may in turn function in maintaining F-actin cytoskeleton and cellular morphology. Together these findings advance our understanding of the mechanism of the cataract pathology in *TDRD7−/−* lenses. Further, more broadly, they serve to expand the role of TDRD-family proteins in cellular differentiation during organogenesis.

Finally, this research highlights that an integrated multidisciplinary approach based on various animal gene-perturbation models, high-resolution phenotypic characterizations, cell biological methods and state-of-the-art molecular analyses involving RNA-Sequencing (RNA-Seq) and fluorescence two-dimensional difference in-gel electrophoresis (2D-DIGE), in combination with existing information from large databases such as iSyTE, is effective in providing novel insights into regulatory mechanisms in development and pathology of specific tissues.

## Results

### Light microscopy and histology show that loss of *Tdrd7* causes lens defects and fully penetrant cataracts in early postnatal mice

**Figure 1:**
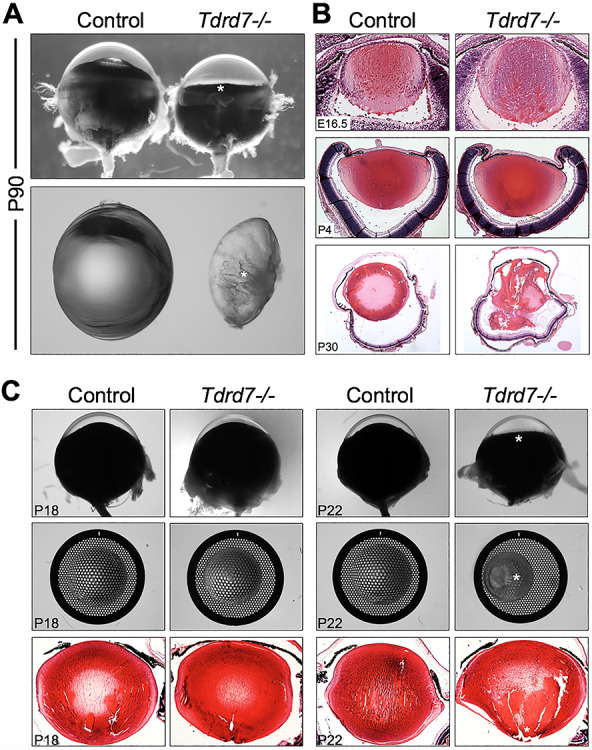
Phenotypic characterization of *Tdrd7−/−* lens defects. (**A**) Light microscopy-based examination of ocular defects in postnatal (P) day 90 *Tdrd7−/−* mouse lenses. The control eye exhibits a normal iris and a clear lens (left panels), while *Tdrd7−/−* eye shows a flattened iris (top right panel, indicated with asterisk) and a smaller lens with posterior rupture and cataract (bottom right panel indicated with asterisk). Note: the dissected lenses in the bottom panel are balanced on their equator. (**B**) Histological analysis of control and *Tdrd7−/−* eye tissue at embryonic (E) 16.5 and postnatal (P) 4 and P30 stages. While the control lenses show no defects through P30, *Tdrd7−/−* lenses exhibit profound lens defects with 100% penetrance at P30. (**C**) *Tdrd7−/−* mice display visible eye and lens defects by P22. Light microscopy of control and *Tdrd7−/−* eye and lens indicates no discernable difference at P18. However, at P22, *Tdrd7*−/− mice exhibit a flattened iris (asterisk) and cataract (asterisk, right image middle panel) at 100% penetrance. Furthermore, lens defects in *Tdrd7*−/− mice are also detected by histological analyses at P22.

We focused on a detailed characterization of lens cataracts in *Tdrd7−/−* mice that were generated as a targeted germline knockout carrying a deletion spanning exons 8–12, as described previously (32). To gain insight into the etiology of *Tdrd7−/−* cataracts, the eyes from these animals were examined using light microscopy and histology. These analyses validated that the *Tdrd7−/−* mice exhibit profound ocular and lens defects at age 3 months (Fig. 1A). Next, to identify the earliest age when the ocular phenotypes are first evident, we analyzed control and *Tdrd7−/−* mouse lenses at several embryonic (E) and postnatal (P) days ranging from E16.5 to P30. While histological analyses indicate no obvious defects at embryonic (E16.5) or early postnatal (P4) stages, by stage P30, *Tdrd7−/−* lenses have profound defects (Fig. 1B). To narrow the window of the onset of the defects further, bright-field microscopy and grid imaging were performed. While at P18 there are no obvious lens or eye defects in *Tdrd7−/−* mice, by P22 cataracts are detected in the knockout animals with 100% penetrance (Fig. 1C). Although histological analysis also detects subtle lens defects in *Tdrd7−/−* mice at P22 (Fig. 1C), these abnormalities become more severe with age (e.g. P30) (Fig. 1B). These data demonstrate lens defects discernable by light microscopy that present precipitously between stages P18 and P22 in *Tdrd7−/−* mice. These findings suggest that *Tdrd7* deficiency leads to a progressive buildup of pathological changes in lens cells, which, after crossing a threshold, within a few days manifests as cataracts.

### RNA-Seq analysis identifies differentially expressed genes in *Tdrd7−/−* lenses

**Figure 2:**
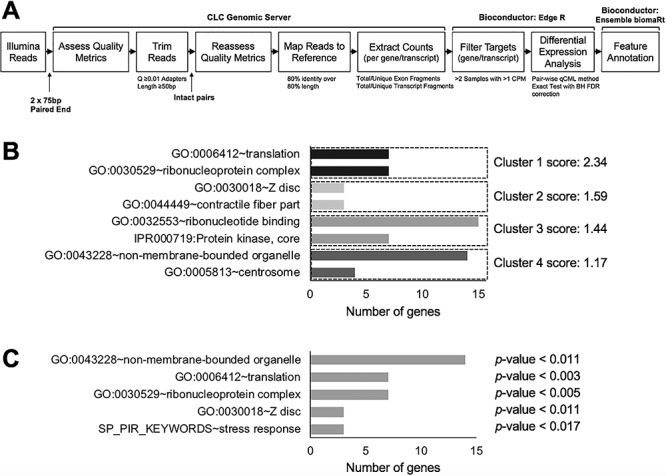
RNA-Seq analysis of *Tdrd7−/−* lens. (**A**) Bioinformatics pipeline for analyzing RNA-Sequencing data; delineating various steps for quality control, adaptor removal, sequence integrity, sequence mapping, estimating counts per transcript and differential analysis. (**B**) Gene ontology (GO) analysis of 49 differentially expressed genes with fold change (FC) >1.5 and FPKM >10 expression in lens tissue. (**C**) GO analysis of 85 differentially expressed genes with FC >1.5 and FPKM > 5 expression in lens tissue. (C) GO analysis of 168 differentially expressed genes with FC >1.5 and FPKM > 2 expression in lens tissue.

To gain insights into the molecular basis of the pathology in *Tdrd7−/−* lens, genome-level transcript profiling by RNA sequencing (RNA-Seq) was performed. To minimize the detection of potential secondary alterations in RNA-Seq transcript profiles in *Tdrd7−/−*, stage P4 lenses were selected, as this stage is 18 days prior to detection of the cataract by light microscopy. An established pipeline was used for initial processing and analysis of RNA-Seq data (Fig. 2A). This analysis identified a total of 280 differentially expressed genes (DEGs), including 123 up- and 157 downregulated genes, at 1.5-fold change cutoff and <0.05 significant *P*-value (Supplementary Table S1). A heat map of the top 100 up- or downregulated genes in the context of their expression or enriched expression in normal lens development indicated that both up- or downregulated genes were similarly enriched for lens-enriched genes (Supplementary Fig. S1). *Tdrd7−/−* lens DEGs were first subjected to cluster analysis using the Database for Annotation, Visualization and Integrated Discovery (DAVID) bioinformatics tool (https://david.ncifcrf.gov/) to identify genes with common functions or those commonly involved in specific pathways (36, 37). This analysis identified gene ontology (GO) clusters for categories ‘translation’, ‘ribonucleoprotein complex’, ‘Z-disc’, ‘contractile fiber part’, ‘ribonucleotide binding’ ‘protein kinase, core’, ‘non-membrane-bound organelle’ and ‘centrosome’ (Fig. 2B, Supplementary Table 2). This analysis also identified gene ontology (GO) charts for categories ‘translation’, ‘ribonucleoprotein complex’, ‘Z-disc’, ‘ribonucleotide binding’, ‘non-membrane-bound organelle’ and ‘stress response’ (Fig. 2B, Supplementary Table 3). ‘Z-disc’ and ‘contractile fiber’ categories also shared genes with ‘cytoskeleton’.

### iSyTE-based analysis of RNA-Seq data identifies high-priority candidate genes associated with *Tdrd7−/−* lens defects

**Figure 3:**
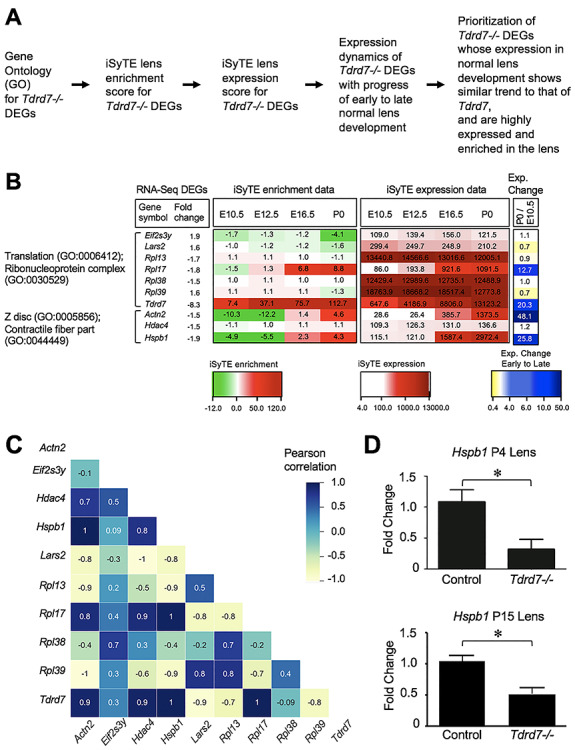
iSyTE-based analysis of *Tdrd7−/−* lens RNA-Seq data identifies *Hspb1* among the high-priority candidates. (**A**) Flowchart of analysis of RNA-Seq data for the identification of high-priority candidate genes in *Tdrd7−/−* lens. (**B**) An integrated analysis using iSyTE lens expression and enrichment data along with functional annotation and GO enrichment of significant (FC ±1.5, *P*-value ≤ 0.05) RNA-Seq differentially expressed genes (DEGs). GO categories for DEGs are indicated on the left. iSyTE-based expression and enrichment data at normal embryonic and postnatal lens development stages (E10.5, E12.5, E16.5 and P0) for the *Tdrd7−/−* lens DEGs are represented in the heat map diagram using ‘red–white–green’ and ‘red–white–white’ color gradients, respectively. Lens expression of DEGs at early postnatal stage P0 over that at early embryonic stage E10.5 (expression in late versus early lens) is represented using ‘blue–white–yellow’ color gradient. (**C**) Pearson correlation heat map of candidate genes based on iSyTE wild-type lens expression data across the four developmental stages. The integrated approach outlined in (A–C) applies GO category enrichment as well as iSyTE-based analysis of DEGs and associates Hspb1 as a top priority candidate gene (criteria: expression score in normal lens >2000, enrichment score in normal lens > 4.0, differential expression in *Tdrd7−/−* lens >1.5-fold change, *P*-value <0.05). (**D**) RT-qPCR analysis of *Hspb1* validates its downregulation in *Tdrd7−/−* lenses at stages P4 and P15. Asterisk indicates *P*-value <0.01.

Next, to prioritize candidate genes from *Tdrd7−/−* lens list of DEGs that may be of significance to the cataract pathology, we further analyzed the GO-enriched genes using a custom strategy (Fig. 3A) involving normal lens developmental gene expression and enrichment data in iSyTE (38, 39). This analysis identified several *Tdrd7−/−* misregulated genes from the top scoring GO categories ‘translation’ or ‘ribonucleoprotein complex’ and ‘Z-disc’ or ‘contractile fiber part’ (Fig. 3B). Among these DEGs, while all were significantly expressed in normal lens development, the candidates *Actn2*, *Hspb1* and *Rpl17* exhibited high lens-enriched expression as per iSyTE analysis (Fig. 3B). Furthermore, GO categories with lower scores were identified as ‘protein kinase core’, ‘ribonucleotide binding’, centrosome and ‘non-membrane-bound organelle’ (Supplementary Fig. S2). These included other candidates that also exhibit high lens-enriched expression in normal development. These were *Atp13a5*, *Dcakd*, *Cbx4*, *Clic5*, *Frmpd1*, *Mylk*, *Poln* and *Usp2* (Supplementary Fig. S2). However, among these various candidates with lens-enriched expression, Hspb1 was the only candidate that had the highest expression in the lens (Fig. 3B) and met all the criteria of expression score in normal lens >2000, enrichment score in normal lens >4.0 and differential expression in *Tdrd7−/−* lens >1.5-fold change (*P*-value <0.05). To further prioritize these DEGs, we investigated which of these candidates exhibit high correlation with *Tdrd7* expression pattern in normal lens development. The change in Hspb1 expression at stages E10.5 and P0 was comparable to the change in Tdrd7 (Fig. 3B). To extend this analysis for the expression of all candidate genes across all the four stages in normal lens development, we performed the Pearson correlation. The Pearson correlation analysis showed that *Hspb1* was among the top two genes that exhibited high positive correlation with *Tdrd7* expression in normal progression of lens development (Fig. 3C). HSPB1 is involved in controlling cellular F-actin in cells under stress conditions (40). Therefore, we next performed RT-qPCR analyses and validated the RNA-Seq observations that *Hspb1* mRNA is reduced in *Tdrd7−/−* lenses (Fig. 3C). Thus, together these analyses identified *Hspb1* as a high-priority candidate gene in *Tdrd7−/−* lens RNA-Seq DEGs.

### 2D-DIGE analysis independently identifies HSPB1 protein to be reduced in *Tdrd7−/−* lens

**Figure 4:**
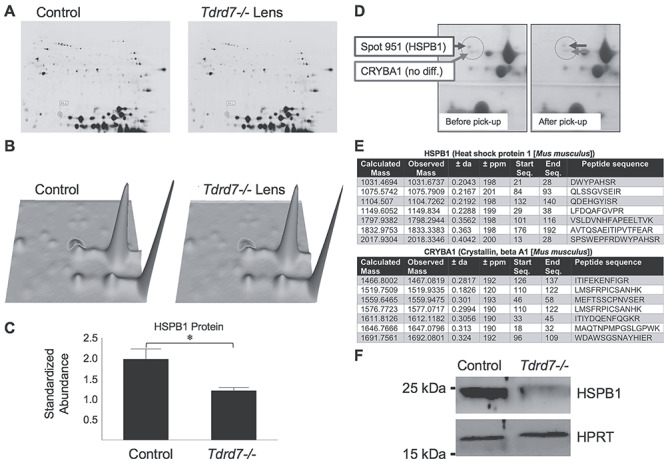
2D-DIGE proteome screen identifies HSPB1 protein to be downregulated in *Tdrd7−/−* lens. (**A and B**) 2D-DIGE analysis showed that protein coinciding with ‘spot 951’ was downregulated in *Tdrd7−/−* lens. (**C**) Abundance of spot 951 (later identified as HSPB1 protein) was significantly reduced in P15 *Tdrd7−/−* lens (asterisk represents *P*-value of 0.039). (**D**) Spot 951 and a reference spot (later identified as CRYBA1) was picked up for LC-MS/MS-based identification. (**E**) LC-MS/MS identified spot 951 as mouse HSPB1 with a 100% protein CI as well as a 100% Best Ion CI, while the reference spot was identified as CRYBA1. (**F**) Western blot confirmed the reduction of HSPB1 protein in *Tdrd7−/−* lenses compared to control.

In addition to RNA-Seq, a global proteomics-based approach was taken to uncover potential alterations in the *Tdrd7−/−* lens proteome. Fluorescence two-dimensional difference in-gel electrophoresis (2D-DIGE) was performed on *Tdrd7−/−* lenses to screen for changes in protein expression caused by the loss of TDRD7. Previous efforts have demonstrated that the lens proteome is dominated by the presence of high levels of crystallin proteins (41, 42), making the identification of quantifiable differences in non-crystallin proteins between control and *Tdrd7−/−* lenses challenging. Despite these caveats, any protein alterations that are detected are likely indicative of their significance in the manifestation of the cataract phenotype. Similar to RNA-Seq, to minimize detection of potential secondary alternations, 2D-DIGE analysis was performed at P4, prior to the earliest stage (i.e. stage P18; Fig. 1) when phenotypic changes are first detected in the *Tdrd7−/−* lens. Considering that it may take longer for protein level changes to be reflected in the knockout lens, we also selected stage P15, which is temporally closer to the first stage of the detection of the phenotype, for this analysis. This strategy was expected to identify any significant protein changes that likely contributed to the pathology of *Tdrd7−/−* lenses. This analysis detected a single protein to be significantly reduced in *Tdrd7−/−* lens compared to control at 1.6-fold change (Fig. 4A–C). The differentially expressed protein was of relatively low molecular weight (~25 kDa), similar to the molecular weight of crystallins, but not as abundant. The protein from this spot was isolated from the gel and subjected to LC-MS/MS analysis (Fig. 4D), which identified it as the heat shock protein HSPB1 (Fig. 4E). HSPB1 protein reduction in *Tdrd7−/−* lens was independently validated by western blotting (Fig. 4F). Thus, the 2D-DIGE screen independently identified HSPB1 to be downregulated in the absence of TDRD7 in the lens, further validating the RNA-Seq data.

### High-resolution phenotypic analysis by scanning electron microscopy shows severe lens fiber cell defects in *Tdrd7−/−* mice prior to overt cataract formation

**Figure 5:**
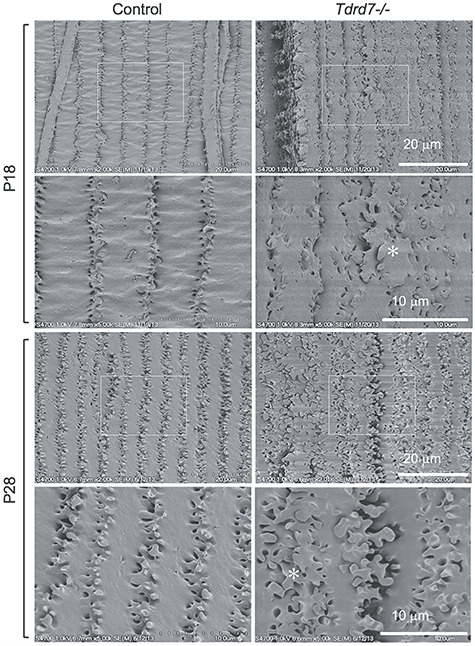
Scanning electron microscopy demonstrates *Tdrd7−/−* lenses have abnormal fiber cell morphology. Scanning electron microscopy (SEM) was performed to visualize cortical fiber cells for control and *Tdrd7−/−* lenses at stages P18 and P28. While the control appeared normal, abnormal fiber cell morphology (asterisk) was observed in *Tdrd7−/−* lenses at both P18 and P28.

The molecular characterization of *Tdrd7−/−* mouse lenses by RNA-Seq followed by rigorous bioinformatics analysis as well as 2D-DIGE analysis led to the identification of the high-priority TDRD7 target candidate, *Hspb1*, in the lens. Since HSPB1 is implicated in the modulation of F-actin in cells under stress (40), we next performed scanning electron microscopy (SEM) as high-resolution phenotyping to investigate cellular morphology-based defects in *Tdrd7−/−* lenses. SEM was performed on *Tdrd7*−/− and control lenses at two different postnatal stages, prior to and after detection of overt cataracts. SEM of lenses at stage P18, prior to overt cataract detection, shows that the *Tdrd7−/−* cortical lens fiber cell morphology is not uniform and cells exhibit variably sized and shaped membrane protrusions (Fig. 5). At P28, *Tdrd7−/−* lens fiber cell membrane protrusions appear to be further malformed, appearing very pleomorphic in comparison with control (Fig. 5), which is consistent with the appearance of cataracts. These data indicate that the onset of fiber cell morphology and organizational defects in *Tdrd7−/−* lens is initiated prior to manifestation of severe opacities that are discernable by light microscopy and anatomical phenotyping approaches, such as histology.

### *Tdrd7−/−* mice exhibit abnormal morphology specifically in lens fiber cells that have undergone nuclear degradation

**Figure 6:**
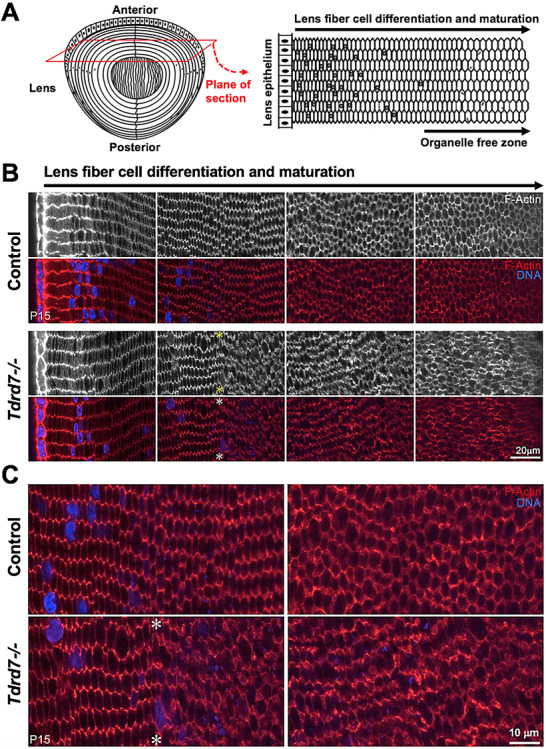
Phalloidin staining for F-actin demonstrates abnormal cellular morphology specifically in fiber cells after nuclear degradation in *Tdrd7−/−* lenses. (**A**) Diagram of a lens (left) with the cross-sectional plane (red) through the lens equator. Schematic of the resulting lens equatorial cross section (right) wherein epithelial cells are located at the periphery and hexagonal morphology of fiber cells is visualized. Early to late maturation stages of differentiating fiber cells are indicated, along with the organelle-free zone. (**B**) Control and *Tdrd7−/−* lens sections at P15 were stained with phalloidin to visualize F-actin. DNA was visualized by Hoechst stain. Images from comparable areas representative of early to late fiber differentiation and maturation (left to right) are shown for control and *Tdrd7−/−* lenses. There is no discernable difference in young differentiating and maturing lens fiber cells (left-most panel and left-half of the second panel) between control and *Tdrd7−/−* lenses. However, coinciding with nuclear degradation (asterisk), fiber cells in *Tdrd7−/−* lens showed abnormal F-actin distribution, indicative of abnormal fiber cell morphology as compared to control (second panel). (**C**) Zoom-in of middle image panels shows abnormal fiber cell morphology and F-actin distribution coinciding with nuclear degradation (asterisk) and beyond in *Tdrd7−/−* lens.

**Figure 7:**
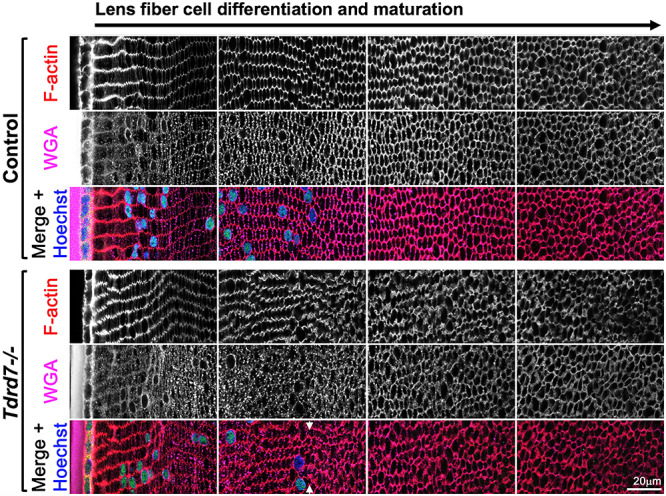
WGA and F-actin staining demonstrates abnormal membrane morphology and fiber cell organization specifically in fiber cells with nuclear degradation in *Tdrd7−/−* lenses. Control and *Tdrd7−/−* lens sections at mouse stage P15 were stained with wheat germ agglutinin (WGA) and phalloidin to visualize cellular membrane and F-actin, respectively. Hoechst stain was used to visualize DNA. Images from comparable areas representative of early to late fiber differentiation and maturation (left to right) are shown for both control and *Tdrd7−/−* lenses. While there is no discernable difference in young differentiation and maturing lens fiber cells at the lens periphery (left-most panel and left-half of the second panel) between control and *Tdrd7−/−* lenses, fiber cells showed abnormal WGA staining and abnormal fiber cell morphology in the *Tdrd7−/−* lens, coinciding with nuclear degradation (arrowheads), compared to control (second panel).

Based on the morphological defects identified by SEM, we next undertook the characterization of the fiber cell cytoskeleton in *Tdrd7−/−* lens. We examined *Tdrd7−/−* lenses in equatorial cross sections to allow for visualization of fiber cell hexagonal architecture and packing at different stages of differentiation and maturation, from newly formed cortical fiber cells (closest to the epithelial cells) to mature fiber cells after organelle degradation, which is marked by nuclear degradation. Initially, we evaluated F-actin in control and *Tdrd7−/−* lenses, since F-actin is associated with fiber cell membranes and plays an important role in controlling fiber cell organization and morphology (43). Phalloidin staining for F-actin showed that compared to control lenses that exhibit a normal hexagonal cellular pattern, *Tdrd7−/−* lenses exhibit abnormalities in the hexagonal cell morphology and packing, particularly in cells that have undergone or are undergoing nuclear degradation and are entering late stages of maturation (i.e. organelle-free zone, OFZ) (Fig. 6A and B). We also stained fiber cell membranes using wheat germ agglutinin (WGA), which is a lectin that binds to sialic acid and N-acetyl-D-glucosaminyl residues on cell membranes (44). WGA staining demonstrated that *Tdrd7−/−* lenses exhibit abnormal fiber cell membrane morphology starting in the zone of nuclear degradation (Fig. 7). This indicates that abnormal fiber cell membrane morphology contributes to abnormal appearance of F-actin staining in the *Tdrd7−/−* lenses. Together, these data show that mature fiber cells with nuclear degradation need TDRD7 to maintain proper morphology and regular hexagonal packing pattern.

### Immunostaining shows HSPB1 protein is reduced in *Tdrd7−/−* lens fiber cells

**Figure 8:**
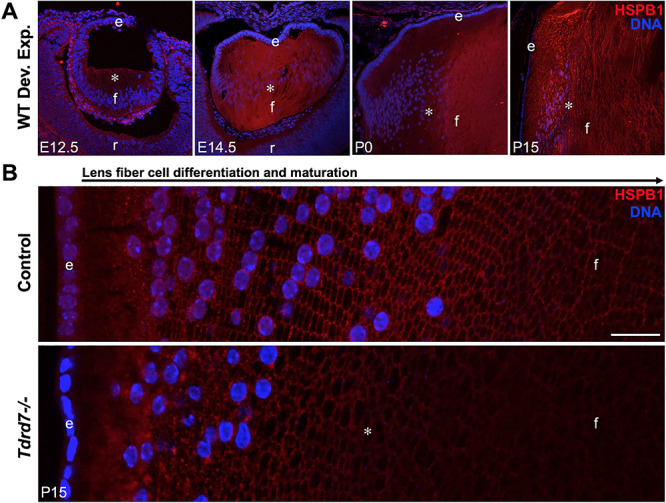
HSPB1 protein staining in fiber cells increases during normal lens development but is reduced in *Tdrd7−/−* lens fiber cells. (**A**) To examine HSPB1 protein expression in normal mouse lens development, immunostaining with HSPB1 antibody was performed on sagittal sections of wild-type mouse embryonic lenses E12.5 and E14.5 and postnatal lenses at P0 and P15. HSPB1 is progressively expressed in lens fiber cells during development (asterisk). (**B**) Cross sections of control and *Tdrd7−/−* P15 lenses were immunostained with HSPB1 antibody. Reduction in HSPB1 protein staining in *Tdrd7−/−* fiber cells that have lost their nuclei is indicated by asterisk. DNA (blue) is stained by DAPI. Scale bar indicates 20 μm. Abbreviation: e, anterior epithelium of the lens; f, fiber cells; r, retina.

Because HSPB1 was found in multiple screens of gene and protein expression changes in *Tdrd7−/−* lenses, and it is an F-actin modulator that may help explain the fiber cell morphological defects, we next focused on characterization of HSPB1 expression and localization in lenses with respect to lens development, fiber cell differentiation and maturation. While western blotting showed reduced HSPB1 protein in *Tdrd7−/−* whole lenses (Fig. 4A), it does not provide spatiotemporal information on its distribution in normal or *Tdrd7−/−* lenses. First, we investigated HSPB1 protein expression in normal lens development by immunostaining of lens sagittal sections from E12.5 to P15. Immunostaining shows that at E12.5, HSPB1 staining is detected weakly in the primary differentiating fiber cells, while at E14.5, HSPB1 staining increases beginning with secondary fiber cell differentiation (Fig. 8A). At P0, there appears to be elevated HSPB1 staining in the fiber cells in the center of the lens, possibly indicating relatively increased protein expression in late fetal lens fiber cells, which form the centrally located fiber cells of the P0 lens. However, by P15, HSPB1 staining is present fairly uniformly throughout the lens, both in the differentiating fiber cells at the lens periphery and in the inner, more mature fiber cells after they lose their nuclei (Fig. 8A). The analysis of P15 lens cross sections at higher magnification shows that staining for HSPB1 protein appears to be elevated in the inner cortical fiber cells where it is associated with fiber cell membranes and remains associated with the fiber cell membranes even after they have lost their nuclei (Fig. 8B, top panel). By contrast, in *Tdrd7−/−* lens cross sections, HSPB1 protein staining appears reduced in the mature lens fiber cells after they have lost their nuclei (Fig. 8B, lower panel).

### RNA immunoprecipitation shows that TDRD7 protein associates with *Hspb1* mRNA

**Figure 9:**
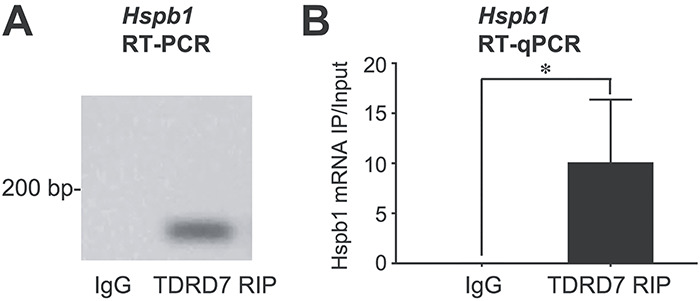
TDRD7 protein associates with *Hspb1* mRNA in the lens. RNA immunoprecipitation (RIP) analysis using TDRD7 antibody was performed for wild-type mouse lenses at stage P15. This was followed by (**A**) RT-PCR and (**B**) RT-qPCR analysis, both of which demonstrate that *Hspb1* mRNA is enriched in TDRD7 pulldowns compared to control. Asterisk indicates *P*-value <0.05.

TDRD7 is known to closely associate with RNA in ribonucleoprotein complexes such as RNA granules (17, 30, 32, 45). Therefore, we next investigated if the altered levels of *Hspb1* mRNA and consequently HSPB1 protein in *Tdrd7−/−* lenses are a result of disruption of a potential interaction between TDRD7 protein and *Hspb1* mRNA in a ribonucleoprotein complex in normal lens development. To test this hypothesis, we performed RNA immunoprecipitation (RIP) assays on wild-type mouse lens lysates at P15 using TDRD7-specific antibody followed by a semiquantitative and RT-qPCR analysis of *Hspb1* mRNA. RIP assays demonstrate a significant enrichment of *Hspb1* mRNA in TDRD7-RIP pulldown but not in the IgG-only control (Fig. 9). These biochemical analyses suggest that TDRD7 is closely associated with *Hspb1* mRNA in the lens.

### Single-molecule fluorescence *in situ* hybridization shows that TDRD7 protein co-localizes with *Hspb1* mRNA in differentiating lens fiber cells

**Figure 10:**
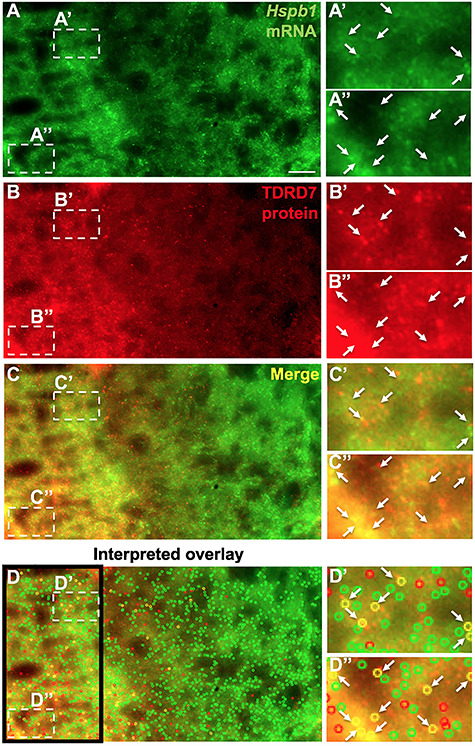
Single-molecule fluorescence *in situ* hybridization (smFISH) coupled with immunostaining demonstrates TDRD7 protein to co-localize with *Hspb1* mRNA in lens fiber cells. (**A**) Wild-type mouse lens at stage P15 was stained with complementary RNA probes specific to *Hspb1* mRNA (green), (**B**) along with immunostaining for TDRD7 protein (red). (**C**) Merged image of the co-staining (yellow) and (**D**) analysis of significant co-localization of *Hspb1* mRNA and TDRD7 protein using custom-written MATLAB program as described in Methods (colored open circles). (A’-D”) shows zoom-in of regions indicated by broken-line boxes in A–D. Arrows indicate co-localizing elements scored by the MATLAB analysis.

While RIP assays suggest a close association of TDRD7 protein with Hspb1 mRNA, it does not inform whether these associations occur *in vivo*. Therefore, we examined the relevance of these biochemical interactions in the context of fiber cell biology in lens development. In order to directly visualize the association of TDRD7 protein with its target mRNA, we used the state-of-the-art single-molecule RNA imaging technique, single-molecule fluorescence *in situ* hybridization (smFISH) (46, 47), coupled with immunostaining to image these interactions at single-molecule resolution. We performed this analysis on wild-type mouse P15 lens sections. This analysis demonstrated that TDRD7 protein co-localized with *Hspb1* mRNA in differentiating lens fiber cells (Fig. 10). Image analysis for co-localization between protein and RNA using custom-written programs in MATLAB (48, 49) indicated that about 10% of the TDRD7 protein is co-localized with *Hspb1* mRNA in the examined area of late differentiating fiber cells prior to nuclear degradation (Fig. 10). These data indicate that TDRD7–ribonucleoprotein (RNP) complexes involving specific target mRNAs, such as *Hspb1*, may be necessary for optimal mRNA and protein levels in the lens, which in turn is necessary for generating normal cellular morphology in differentiating lens fiber cells.

### *Hspb1* knockdown in *Xenopus* causes eye and lens defects

**Figure 11:**
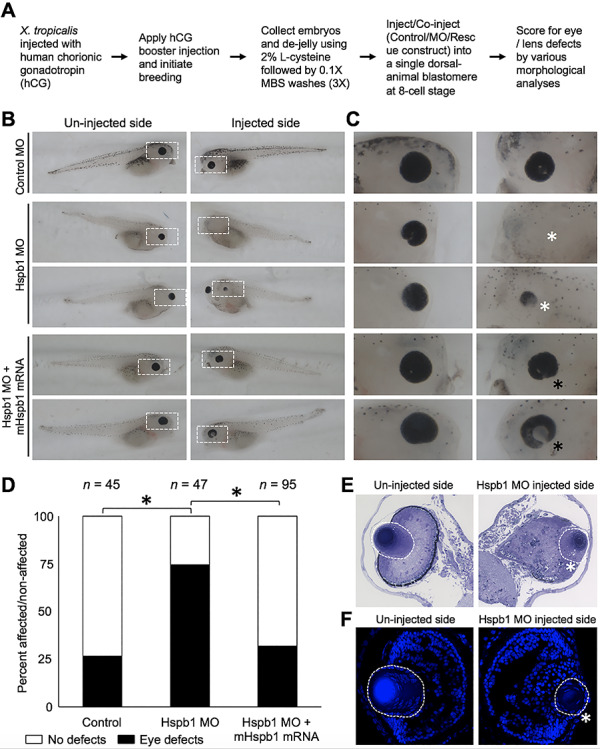
*Hspb1* knockdown in *X. tropicalis* causes eye and lens defects. (**A**) Flowchart of *X. tropicalis* assay and phenotypic scoring. (**B**) Representative images of un-injected and injected side of *X. tropicalis* embryos injected with either control MO, Hspb1 MO or Hspb1 MO together with mouse Hspb1 mRNA (mHspb1). (**C**) Zoom-in images of white-dotted area in (B). (**D**) Chart showing the distribution of ocular defects in *X. tropicalis* embryos injected with either control MO, Hspb1 MO or Hspb1 MO together with mHSPB1 mRNA. The number of embryos for the different conditions is indicated on top. Black asterisk represents significant differences in the observed number of eye defects between the different conditions. For the control MO versus Hspb1 MO comparison, the *P*-value is 4.54 × 10^−6^, and for the Hspb1 MO versus Hspb1 MO + mHspb1 mRNA comparison, the *P*-value is 1.38 × 10^−6^. The control MO versus Hspb1 MO + mHSpb1 mRNA comparison is not significant (*P*-value = 0.55). (**E**) Histology analysis-based images and (**F**) DAPI-stained images of sections of un-injected and Hspb1 MO injected *X. tropicalis* embryos. White asterisk represents small eye/lens defects in Hspb1 MO-injected side of embryos. Black asterisks in (C), (E) and (F) represent rescue of eye defects in Hspb1 MO + mHspb1 mRNA-injected side of embryos. In (E) and (F), the lens is indicated by a white-dotted area.

We next sought to further investigate the connection of HSPB1 with eye/lens development and defects. To examine the impact of Hspb1 deficiency on eye and lens development, we used morpholinos to develop an *Hspb1*-knockdown model in *Xenopus tropicalis* (Fig. 11A). While the control morpholino had no effect, the *Hspb1* morpholino-injected side of stage 42 *X. tropicalis* tadpoles exhibited eye and lens defects, and the un-injected side did not (Fig. 11B). Importantly, these ocular defects in *Xenopus* were rescued by a simultaneous injection of mouse *Hspb1* mRNA (Fig. 11B and C). Hspb1 knockdown led to the reduced size of the lens (Fig. 11D–F). Chi-squared analysis of the affected tadpoles versus non-affected tadpoles from both the Hspb1 morpholino-injected and un-injected sides showed that the observed ocular defects due to *Hspb1* knockdown were significant (*P* < 0.05). Together, these data indicate that Hspb1 is important for eye and lens development.

## Discussion

The bulk of the mammalian lens is made of terminally differentiated fiber cells. To ensure that the lens is transparent for optimal passage of light, fiber cells undergo degradation of nuclei and other organelles (33, 34) while acquiring/maintaining an unusually elongated cellular morphology and being precisely aligned with adjacent cells. Due to this ‘extreme’ form of differentiation, fiber cells that reside in the ‘organelle-free zone’ of the lens are transcriptionally and translationally ‘silent’ (50, 51). Thus, the question arises as to what are the mechanisms by which these cells, in the absence of active transcription and translation, coordinate their key factors in order to maintain their characteristic morphology. Specifically, what are the mechanisms that fiber cells use to ensure that F-actin and other cytoskeletal proteins are maintained while other proteins and organelles are being degraded? The findings in the present study address these questions.

Recent data indicates that post-transcriptional regulatory factors have a key role in controlling fiber cell differentiation and their misregulation is implicated in the development of cataract (17–21, 52–54). Specifically, the finding that eye/lens defects or cataracts can be caused by functional compromise of post-transcriptional regulatory proteins such as TDRD7, CELF1, RBM24 or CARPIN2 was unanticipated since the majority of prior known cataract-causing mutations disrupted lens structural proteins or transcription factors (2, 12, 14, 16, 20, 55–57). However, compared to transcription factors and signaling molecules, our understanding of post-transcriptional regulatory proteins and the pathways they control in development, in general, and in lens development, in particular, remains limited (16, 20, 58, 59). To address this deficit and gain insight into the molecular and cellular basis of the cataract pathology that arises from TDRD7 deficiency, in the present study, we have applied diverse approaches at the phenotypic, cellular, molecular and system levels to investigate the cataract defect in *Tdrd7−/−* mice, which closely phenocopies congenital cataract in humans.

**Figure 12:**
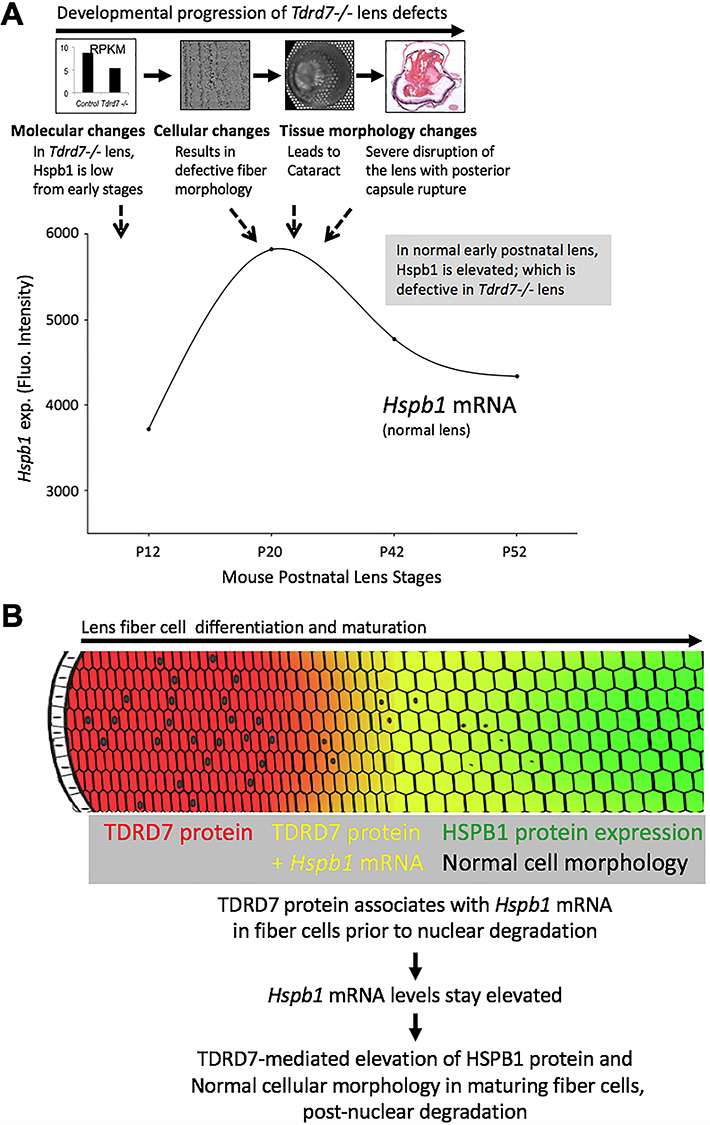
Models for TDRD7 function in lens development and cataractogenesis. The following models are proposed to explain the temporal and the cell and molecular basis of the lens defects and cataract observed in *Tdrd7−/−* mice. (**A**) Overlay of temporal aspects of *Tdrd7−/−* lens defects, at a molecular and phenotypic level, corresponding to wild-type expression of the TDRD7 target mRNA *Hspb1*, a heat shock protein associated with F-actin under stress conditions, in normal mouse lens development. iSyTE microarray-based expression analysis of wild-type lenses shows that mRNA expression of *Hspb1* upregulates before slight downregulation, but yet maintaining relatively high levels in postnatal wild-type lens development. Interestingly, *Tdrd7−/−* lenses show reduced levels of *Hspb1* mRNA at early postnatal stages (RNA-Seq, RT-qPCR data), several days prior to morphological detection of the lens cellular defects. Furthermore, HSPB1 protein levels remain low in *Tdrd7−/−* lenses in later postnatal stages at P15 (proteome, western, immunofluorescence data), just prior to the initial detection of the defect at the cellular level (SEM at P18) or the gross morphological level (overt cataract at P22). The timing of expression of the lens defects in *Tdrd7−/−* mice coincides with the timing of normal upregulation of *Hspb1* in wild-type postnatal lens development and therefore may reflect the disruption of the expression dynamics of this F-actin-associated protein upon TDRD deficiency. (**B**) Based on the current molecular and phenotypic data, including the critical observation that *Tdrd7−/−* mice show abnormal cellular morphology particularly in fiber cells after nuclear degradation, the following model is proposed. The association of TDRD7 protein with *Hspb1* mRNA ensures its elevated levels, which translate into high levels of HSPB1, a heat shock molecular chaperone protein necessary for F-actin stability in cells subjected to stress. TDRD7-based elevated levels of HSPB1 protein may function in the maintenance of normal F-actin distribution and cellular morphology in late maturation stage fiber cells, which can be considered a stress-like condition as they undergo nuclear degradation.

A detailed phenotypic characterization of *Tdrd7−/−* mouse lenses at different stages using various approaches revealed that the gross morphology and transparency of knockout or control lenses are indistinguishable at P18. However, at P22, just 4 days later, all *Tdrd7*−/− mice examined (*n* > 50) exhibit severe lens defects, suggestive of a global breakdown of cellular morphology by this stage. In the past, we had applied microarray-based gene expression profiling to examine *Tdrd7* null lenses, which were obtained from mice identified in an ENU mutagenesis screen (17). In the present study, we applied a high-throughput approach, RNA-Seq, to gain insight into the molecular changes in *Tdrd7−/−* (targeted knockout) mouse lenses. Importantly, we selected an early postnatal stage, P4, for this analysis because it is 14 days prior to the earliest stage that lens cellular morphological changes are detectable by SEM in *Tdrd7−/−* mice. This approach increases the possibility for detecting the earliest molecular changes that are likely associated with the lens defects. To prioritize promising candidates, we applied an integrated approach to the analysis of the RNA-Seq data involving the identification of the DEGs that exhibit iSyTE lens expression or enrichment as well as a developmental expression pattern similar to that of TDRD7 in the lens. This analysis identified, among the top candidates, *Hspb1* (*Hsp27*), which we also independently identified in an unbiased 2D-DIGE proteomics screen as a key factor that is significantly reduced in *Tdrd7−/−* lens. Interestingly, iSyTE data shows that *Hspb1* expression in wild-type lens development is enhanced in the embryonic stage (E16.5) when nuclear degradation is initiated (60) and is even more sharply upregulated between postnatal stages P12 and P20, before becoming slightly reduced, but yet maintained at relatively high levels at P42 and later stages (Fig. 12A). This coincides with the time frame when fiber cell defects are observed by SEM at P18 and by gross microscopy and histology at P22 and later stages (Fig. 12A). These findings lead to the model that upregulation of *Hspb1* expression in early postnatal stages—which precedes *Tdrd7−/−* lens defects—is critical for normal lens development. Thus, the disruption of this ‘normal’ *Hspb1* upregulation, as a result of TDRD7 deficiency, may contribute to the lens defects in *Tdrd7−/−* mice (Fig. 12A).

While these findings serve to explain the temporal aspect of cataract in *Tdrd7−/−* mice, we sought to gain insight into the cellular basis of lens defects. The TDRD7 target HSPB1 belongs to the α-crystallin family of small heat shock proteins, which have been proposed to prevent protein aggregation in the lens or, under stress conditions, by sequestering of misfolded/denatured proteins (61, 62). Moreover, α-crystallin proteins are associated with fiber cell cytoskeleton, in particular with actin in the lens—a property that can be disrupted by specific mutations (63–69). Analogously, previous data suggests that HSPB1 (1) may interact with F- or G-actin, (2) is detected in actin-associated protein complexes and (3) is involved in actin cytoskeleton stabilization (70–73). Numerous studies offer support that HSPB1 functions in the maintenance of cytoskeletal integrity under normal or stress conditions (71, 74–78). Further, HSPB1 is also described to interact with the actin-binding proteins, caldesmon, calponin and tropomyosin (70, 79), and can associate with vimentin intermediate filament networks, similar to αB-crystallin (74). Maintenance of fiber cell cytoskeleton is essential for lens transparency, and indeed cytoskeletal proteins, which account for majority of non-crystallin proteins in fiber cells, are central to this process (43, 80).

In light of these numerous connections between HSPB1 and the cytoskeleton, and the importance of latter for lens transparency, we hypothesized that lens fiber cells undergoing organelle degradation in late stages of differentiation can be considered to be in a stress-like condition. This TDRD7 deficiency-led HSPB1 reduction—both in the cytoplasm and membrane—may then result in abnormal F-actin distribution and, in turn, result in defective cell architecture of post-nuclear degradation fiber cells that have lost their nuclei in *Tdrd7−/−* lens. Indeed, our data shows abnormal F-actin staining marking fiber cell membranes in *Tdrd7−/−* lens fiber cells that had undergone nuclear degradation. Staining with WGA independently confirmed abnormal membrane morphology of fiber cells that had undergone nuclear degradation in the *Tdrd7−/−* lens and suggested that alterations in F-actin are related to the abnormal membrane morphology. Specifically, the hexagonal packing pattern of fiber cells in cross section appears altered in *Tdrd7−/−* lenses. Indeed, HSPB1 protein was found to be noticeably reduced in *Tdrd7−/−* lens fiber cells post-nuclear degradation, in contrast to control lenses where HSPB1 protein persists on membranes after nuclear degradation. Further, *Hspb1* knockdown in *X. tropicalis* led to microphthalmia and/or lens defects, and these phenotypes could be effectively rescued by mouse HSPB1. These new data for the first time provide direct evidence of HSPB1’s connection to eye/lens development and defects.

Molecular insights into the nature of the TDRD7–*Hspb1* regulatory relationship were obtained by RNA-IP assays that indicated TDRD7 protein associates with *Hspb1* RNA in the lens. A cellular-/tissue-level relevance of this biochemical interaction was further obtained by visualization of TDRD7 protein and *Hspb1* mRNA co-localization using single-molecule FISH. Besides supporting the findings of the biochemical RNA-IP assays, this analysis demonstrates direct visual evidence that TDRD7 protein associates with *Hspb1* mRNA in fiber cells during lens development, which is likely necessary for optimally high levels of HSPB1 protein in the lens that contribute to proper cell morphology in post-nuclear degradation fiber cells. Together, the present data proposes a model wherein in normal lens development, TDRD7 protein is necessary for optimal levels of HSPB1, which in turn may be involved in F-actin cytoskeletal maintenance of fiber cell membrane morphology and packing organization after nuclear degradation in late maturation stages (Fig. 12B). This may be particularly important in a tissue such as the lens, which expresses extraordinarily high levels of crystallin transcripts, but still need to ensure optimal levels of comparatively lower expressed proteins.

This study opens a new area of research in lens biology through presenting questions such as: what is the nature/outcome of the interaction of the TDRD7 protein complex with *Hspb1* mRNA? Because transcription and translation are severely reduced during apoptosis (81), and lens fiber cell differentiation has paralleled with ‘attenuated apoptosis’ (34, 82, 83), the fiber cells undergoing maturation may have recruited mechanisms for RNP-based stabilization of key mRNAs. Such a mechanism may ensure that even less abundant mRNAs—low expression compared to crystallins—such as *Hspb1*, also get translated into sufficient levels of protein in lens fiber cells. Indeed, cytoplasmic RNA granules/RNP complexes are known to be involved in mediating mRNA stabilization or translation into protein in eukaryotes (84–88). Furthermore, HSPB1 shares homology with crystallins alpha A and alpha B, which are known to be involved in homo- or heterodimers. It can be proposed that the expression of a related protein, HSPB1, allows additional molecular chaperone activity that is needed due to elevated translation and abundance of proteins in the lens. Indeed, HSPB1 has been found to suppress inclusion body formation and sequestering of aggregates of mutant crystallin alpha B (89, 90). However, HSPB1 is also documented to be involved in diverse cellular processes ranging from RNA decay to translational control and may also have RNA-binding properties (91–93). Therefore, in the future it will be interesting to examine the diverse molecular aspects of HSPB1 function in the lens. Finally, in addition to *Hspb1*, RNA-Seq also identified *Actn2* (alpha actinin) among the downregulated genes in *Tdrd7−/−* lens. ACTN2 is an F-actin-binding protein, which in non-muscle cells is involved in facilitating F-actin–membrane interactions and is linked to cardiac defects (94, 95). Our preliminary RT-qPCR data validates *Actn2* as a significantly downregulated gene in *Tdrd7−/−* lens (data not shown). In light of the fiber cell morphological defects observed in *Tdrd7−/−* lens, it will be interesting to further study this protein in the lens.

In summary, the data presented here drives home the point that there are distinct mechanisms—based on pre- or post-nuclear degradation differentiation and maturation stage—for cellular morphology maintenance in fiber cells and that the latter are under downstream control by TDRD7. More generally, these data provide support for the involvement of a TDRD-family protein in controlling expression of a heat shock protein and maintenance of F-actin and cell morphology in cellular differentiation.

## Conclusion

The present study provides significant new insights into the cellular and mechanistic basis of lens defects and cataract pathology. Importantly, while the knowledge that lens fiber cells undergo organelle degradation to become transparent has been known for over 150 years (33), questions remain as to how these cells can selectively prevent breakdown of specific cellular proteins and structures such as the cytoskeleton. These data address this fundamental knowledge gap and provide support for TDRD7 being necessary for optimal levels of HSPB1 as a potential mechanism for maintenance of F-actin cytoskeleton and proper cellular morphology of fiber cells that have undergone organelle degradation. Further, this work offers support for HSPB1’s role in eye and lens development. Thus, along with our recent findings on the involvement of CELF1 in the control of degradation of fiber cell nuclei (21), the present study on TDRD7 serves to demonstrate that different post-transcriptional factors and pathways have evolved distinct functions to control specific aspects of the cellular biology of late differentiation stage fiber cells.

## Materials and Methods

### Animal studies

Animal studies were designed and performed as per the statement issued by the Association for Research in Vision and Ophthalmology (ARVO) for the recommended use of animals in ophthalmic and vision research. The experimental protocols were approved by the University of Delaware Institutional Animal Care and Use Committee (IACUC). *Tdrd7*-targeted germline knockout (KO) mouse line (*Tdrd7^tm1.1Chum^*, hereafter referred to as *Tdrd7−/−*) (32) was used in this study. Mice were housed in a 14-h light to 10-h dark cycle. Mouse-tail tissue genomic DNA was isolated using the Puregene® Genomic DNA Purification Kit (Gentra Systems, Minneapolis, MN). Genotyping was performed using the following PCR primer sets: Tdrd7-WT-g-F 5′-GAG TAA CTC TGG GCG CAG TC-3′, Tdrd7-WT-g-R 5′-GCC ATA GCA ATC AGT GAG CA-3′, expected product size 250 bp; and Tdrd7-KO-g-F 5′ GTC TAA CCC ATT CAG GGA TGA AGA 3′, Tdrd7-KO-g-R 5′ GAA TCC TCA CCA GTT AGC CTC ACC 3′, expected product size 500 bp, as previously described (32). Unless otherwise noted, *Tdrd7+/−* mice, which do not exhibit discernable ocular defects, were used as a control. Wild-type ICR outbred mice (Harlan Laboratories, Frederick, MD) were used for collecting lens tissue in RNA immunoprecipitation (RIP) assays.

### Light microscopy, grid imaging and histology

Mouse whole eye tissue or micro-dissected lenses at several embryonic (E) and postnatal (P) stages were imaged in 1X phosphate-buffered saline (PBS) solution under light- and dark-field optics (Zeiss Stemi SV dissecting microscope). Lenses were imaged on a 200-mesh grid (Electron Microscopy Sciences, Hatfield, PA; Catalog G300H-Cu) in 1X PBS as previously described (96). For histology, mouse eyes at different stages were fixed in 4% paraformaldehyde (PFA) (Acros Organics, Cat. No.: 30525-89-4) for 18 h, washed with 1X PBS for 10 min and stored at 4°C in 70% ethanol prior to paraffin embedding. Paraffin embedding was performed at the Comparative Pathology lab of the College of Agriculture and Natural Resources, University of Delaware. Sagittal sections (5 μm) were prepared, stained with hematoxylin (Electron Microscopy Sciences, Cat. No., 16700) and eosin Y (Fisherfinest, Cat. No., 220-104) (H&E) and imaged using a Nikon camera on an upright Zeiss Axiophot light microscope (Carl Zeiss Microscopy, Thornwood, NY) as previously described (96).

### High-throughput RNA sequencing

For RNA sequencing (RNA-Seq) experiments, mouse lenses at stage P4 (*n* = 15 lenses per biological replicate) were collected from *Tdrd7−/−* or control mice. RNA isolation was performed using the *mir*Vana™ RNA isolation kit (Life Technologies, Grand Island, NY). Total RNA isolation, followed by removal of small molecular weight RNA, was performed according to the manufacturer’s instructions. The RNA library was prepared using TruSeq RNA Library Prep Kit v2 (Illumina), and sequencing was performed on a 2x75 bp paired-end run using standard protocols on an Illumina HiSeq 2500 sequencing system at the University of Delaware Sequencing and Genotyping Center. Briefly, mRNA was purified from the total RNA samples using Oligo dT-conjugated magnetic beads, converted to adaptor-tagged, paired-end fragments which were then used for cluster generation onto a TruSeq v3 flow cell according to the Illumina® TruSeq™ RNA Sample Preparation Kit v2. Sequencing was carried out using the SBS Sequencing Kit. Images were analyzed using the Illumina Pipeline software (version RTA 1.13.48/CASAVA 1.8.2), and bases were called and translated to generate FASTQ sequence files.

### RNA-Seq data analysis

Bioinformatics processing was performed at the University of Delaware Center for Bioinformatics and Computational Biology Core facility. Illumina reads quality was assessed using CLC Genomics Workbench Quality Assessment tool (v. 6.5). Reads were end-trimmed to remove adapter sequence, to obtain a minimum average sequence quality (Q ≥ 0.01) and to allow a maximum of one ambiguous base per read using the Trim Sequences Tool in CLC Genomics Server (v. 5.5). After trimming, reads below 50 bp were discarded, and the remaining intact pairs were mapped to the Mouse reference genome (version GRCm38.73 as downloaded from ENSEMBL) using the CLC Genomics RNA-Seq mapping tool (80% identity over 80% of query). Mapping pairs were compared to the reference annotation to obtain raw counts (unique and total exon reads) for both genes and transcripts (isoform). All targets (genes/transcripts) with a minimum of one count per million in two samples were considered for differential expression analysis. Count data was subjected to differential expression analysis in R/Bioconductor (v. 3.0.1) using the EdgeR package (v. 3.2.4) (97), and *P*-values were adjusted for multiple comparisons using FDR method of Benjamini and Hochberg (1995). Further annotations were linked from ENSEMBL BioMart using the biomaRt R package (v. 2.16.0) (98). Functional annotation clustering analysis of *Tdrd7−/−* RNA-Seq differentially expressed genes (DEGs) was first performed as described (96, 99). Briefly, this analysis used the functional annotation clustering method available on the online tool DAVID (https://david.ncifcrf.gov/summary.jsp; version 6.7). The list of differentially expressed genes in *Tdrd7−/−* lens (5 FPKM expression cutoff, ±1.5-fold change, *P* < 0.05) was submitted as query. From the results output, the top clusters and charts with highest scores and significant *P*-values (scores ranging from 2.3 to 1.17 for cluster analysis and *P* < 0.02 for ‘chart’ analysis) were considered. Some of the redundant gene ontology (GO) and chart categories identified the same set of genes and therefore were considered in the most-relevant representative GO or chart terms. Expression data for *Tdrd7−/−* lens DEGs in normal lens development were estimated from the iSyTE database as previously described (21, 38, 39, 100–102). The heat map showing the Pearson correlation for candidate gene expression across four stages in normal lens development was generated using Seaborn library in Python (v2.7).

### Real-time quantitative RT-PCR

Differentially expressed genes (DEGs) identified by RNA-Seq analysis were independently validated by reverse transcription coupled with quantitative PCR (RT-qPCR), which was performed as follows. Total RNA was independently isolated from three biological replicates from *Tdrd7−/−* or control mice using mirVana™ (Ambion®) or RNAeasy (Qiagen) RNA isolation kit, as described above. cDNA was synthesized from total RNA using Bio-Rad iScript™ cDNA Synthesis Kit (Bio-Rad Laboratories, Hercules, CA), followed by qPCR using RealMasterMix Fast Probe Kit (5 PRIME, Gaithersburg, MD) on ABI 7500 Fast Real-Time PCR System and Software v2.0.3 (Applied Biosystems: Carlsbad, CA). Samples were prepared in a 96-well reaction plate with three biological replicates and at least three technical triplicates. The mean and standard deviations were determined and used to calculate the mean fold change (FC) using log (base 10) transformed data in a nested ANOVA as described (96).

### Fluorescence difference in-gel electrophoresis (DIGE)

Two-dimensional (2D)-DIGE experiments were performed as follows. Lenses from *Tdrd7−/−* and control mice at postnatal stages P4 and P15 (at least three biological replicates) were dissected in 1X PBS, flash frozen and stored at −80°C. Lenses were lysed in DIGE lysis buffer (7 M urea, 2 M thiourea, 4% CHAPS, 30 mM Tris, pH 8.5) and centrifuged. Supernatants were precipitated based on a TCA protein precipitation procedure with an acetone wash (2-D Clean-Up Kit, GE Lifesciences). Samples were resuspended in DIGE lysis buffer and pH was adjusted to pH 8.5. Forty micrograms of each sample was DIGE-labeled using 200 picomole of CyDye per 40 μg of protein (DIGE Minimal Labeling Fluor, GE Lifesciences). A pooled internal standard was generated by combining 10 μg of each sample and DIGE labeling with a separated CyDye fluor. A first-dimension separation, namely, isoelectric focusing, was performed using 24 cm immobilized pH gradient (IPG) strips with a non-linear pH gradient of pH 3.0–10.0 (ReadyStrip IPG Strips, Bio-Rad Laboratories) at 40.5 kVh for 9 h on IPGphor II (Amersham Biosciences). IPG strips were rehydrated in buffer containing 10 μg control or *Tdrd7−/−* protein sample, 8 μg pooled internal sample, 7 M urea, 2 M thiourea, 4% CHAPS, 20 mM DTT and 2% 3–10 pharmalyte. IPGs were equilibrated for 15 min two times in SDS buffer containing 6 M urea, 30% glycerol, 2% SDS, 75 mM Tris–HCl pH 8.8 and either 65 mM DTT (first equilibration) or 135 mM iodoacetamide (second equilibration). This was followed by second-dimension separation that was performed on 24 × 20 cm 10% acrylamide SDE-PAGE gels. Gels were imaged using the Typhoon Trio (GE Lifesciences) and analyzed using DeCyder 2-D Differential Analysis Software (GE Lifesciences). Gels for protein spot pick and isolation were run by rehydrating IPGs in a similar buffer with additional 500 μg or 800 μg of unlabeled P15 lens protein with the same first- and second-dimension procedures. These spot pick gels were stained with Imperial Protein Stain (Thermo Fisher Scientific), and protein spots were matched to previous gels. Protein spot 951 was identified with differential expression in *Tdrd7−/−* lenses compared to control and to a nearby non-differentially expressed lower molecular weight spot. This nearby spot was picked to test the likelihood that spot 951 may represent a post-translational modification of the same protein. Excised spots were processed for identification by mass spectrometry (MS) at the Proteomics and Mass Spectrometry Core Facility at Delaware Biotechnology Institute, University of Delaware.

### Mass spectrometry-based protein identification

Isolated gel spots representing proteins of interest were processed as follows for MS-based identification. Trypsin (Promega) digestion was performed in the presence of reduction (dithiothreitol (Bio-Rad)) and alkylation (iodoacetamide (Sigma)) conditions at 37°C as described (103). This was followed by desalting and concentrating of samples using Ziptips (Millipore). Samples were then applied to a target plate with alpha-cyano-4-hydroxycinnamic acid matrix (Sigma). Data were collected on a 4800 MALDI TOF/TOF Analyzer (ABSciex) in positive ion, reflector mode over a mass range of 850–4000 m/z. Select peaks were further analyzed by tandem mass spectrometry (MSMS) at 1 kV with default calibration. The combined MS and MSMS data were submitted to Mascot v2.4 (Matrix Science) and searched against the NCBI (*Mus musculus* taxonomy), with trypsin specificity, 300 ppm mass tolerance, 0.3 Da MSMS tolerance and variable modifications: for carbamidomethylation (C) and oxidation (M). Proteins were identified based on database matches of ≥99% confidence, including three MSMS matches >99% confidence.

### Western blot analysis

Lenses at stage P15 from *Tdrd7−/−* and control mice were subjected to RIPA lysis buffer to prepare lens lysate. Protein lysate concentration was determined by BCA assay following the manufacturer’s instructions (Pierce BCA Protein Assay Kit, Thermo Fisher Scientific). Equal concentrations of the protein lysate for *Tdrd7−/−* and control lenses were loaded on a 7% polyacrylamide gel and then transferred onto PVDF membrane. The membrane was blocked with 5% milk in Tris-buffered saline with 0.1% Tween 20 (TBST) for 1 h at room temperature, followed by overnight incubation at 4°C with primary antibody for HSPB1 (Abcam, Catalog No. ab2790, used at 1:200 diln.) and for beta-actin (loading control) (Abcam, Catalog No. ab8227) in the blocking buffer. The membrane was then washed and probed with a secondary HRP-conjugated antibody (Cell Signaling, Catalog No. 7074S) for 1 h at room temperature, followed by washes and imaging.

### Scanning electron microscopy

Whole eye tissues from control or *Tdrd7−/−* mice at stages P18 and P28 (*n* = 3 biological replicates) were fixed in a solution of 0.08 M sodium cacodylate buffer pH 7.5 (Electron Microscopy Sciences, Hatfield, PA, USA), 1.25% glutaraldehyde (Electron Microscopy Sciences) and 1% paraformaldehyde (Electron Microscopy Sciences) for 5 h at room temperature. This was followed by dissection of lenses, which were transferred to fresh fixative and incubated for 48 h at room temperature. Lenses were washed in 1X PBS for 10 min. Lenses were peeled using tweezers to expose cortical fiber cells. After peeling, lenses were subjected to serial ethanol dehydration (25, 50, 70 and 100%), with exposure to each dilution for 10 min. Lenses were incubated for 2.5 h in 100% ethanol and then critically point dried using an incubation series of hexamethyldisilazane (Electron Microscopy Sciences) (33%, 66%, 100%, diluted in ethanol). Lenses were mounted on aluminum stubs and coated with gold/palladium for 3 min. Samples were imaged with a field emission scanning electron microscope Hitachi S-4700 (Tokyo, Japan).

### Wheat germ agglutinin and phalloidin staining

Freshly enucleated eyes were collected from P15 *Tdrd7−/−* and control mice. The optic nerve was removed and a small opening made in the posterior of the eye to facilitate fixative penetration. Eyeballs were then fixed for 4 h in fresh 1% paraformaldehyde (PFA) in 1X PBS at 4°C. After fixation, samples were washed in ice-cold 1X PBS, cryoprotected in 30% sucrose, frozen in OCT (optimum cutting temperature) medium (Sakura Finetek, Torrance, CA) and stored at −80°C until sectioning. A Leica CM1950 cryostat was used to collect frozen sections (12 μm thick). Staining of lens sections using rhodamine phalloidin (R415, Thermo Fisher Scientific), wheat germ agglutinin-640R (29026-1, Biotium) and Hoechst 33258 (B2883, Sigma-Aldrich) was conducted as previously described (104, 105). Slides were mounted with ProLong® Gold antifade reagent (Thermo Fisher Scientific), and fluorescence images were collected using a Zeiss LSM780 confocal microscope. Frozen sections in the cross-orientation near the lens equator were identified based on the thickness of the lens epithelium (104). Staining was repeated on three biological replicate samples from different mice for each genotype, and representative data are shown.

### Immunostaining for HSPB1

Eye tissues at stage P15 from *Tdrd7−/−* and control mice were dissected and fixed for 30 min on ice in 4% PFA in PBS. This was followed by a brief wash with 1X PBS and incubation in 30% sucrose overnight at 4°C and embedding in OCT medium. Cryosections were then obtained (12 μm thick) and mounted on microscope-charged slides. Sections were blocked with 5% horse serum and 5% goat serum in PBST (PBS + 0.1% Triton X-100) for 1 h at room temperature. Sections were then incubated with HSPB1 antibody (Abcam ab1426, 1:50 diln) in the blocking buffer overnight at 4°C. Sections were then washed in 1X PBS and incubated with a fluorescently conjugated secondary antibody and Alexa Fluor™-conjugated phalloidin in the blocking buffer for 1 h at room temperature. This was followed by 1X PBS washes, and samples were mounted with VECTASHIELD Antifade Mounting Media (Vector Laboratories, Catalog No. H-1000). Fluorescence images were collected using a Zeiss LSM780 confocal microscope. To assess the potential cross-reactivity with alpha crystallins and evaluate the specificity of HSPB1 antibody, we performed alpha-crystallin staining and compared this with HSPB1 staining in the lens. These revealed different staining patterns for HSPB1 and CryAA (data not shown), indicating that these antibodies do not cross-react and that the immunostaining pattern is specific for HSPB1.

### RNA immunoprecipitation assay

Postnatal day 15 (P15) wild-type mouse ICR lenses (*n* = 20 per biological replicate, total three biological replicates) were freshly dissected and used with the 17-700 Magna RIP™ RNA-Binding Protein Immunoprecipitation Kit (EMD Millipore) according to the manufacturer’s protocol as described (21). This was followed by RT-PCR and RT-qPCR for *Hspb1* mRNA as described above.

### Fluorescence *in situ* hybridization coupled with immunostaining

Eye lens sections from three biological replicates of *Tdrd7−/−* and control at stage P15 were prepared in the cross-orientation at 10 μm thickness to visualize the hexagonal appearance of fiber cells and frozen at −80°C. Sections were fixed with 4% PFA in 1X PBS, permeabilized using 70% ethanol and hybridized with RNA-specific probes. These probes (commercially called as stellaris probes) were a mix of about 40 20-nucleotide-long oligomers, each having the same single fluorophore tag conjugated to its 3′ end. These probes bind in a sequence-specific manner to the target RNA (in this case, mouse *Hspb1*) giving diffraction-limited spots that can be visualized using fluorescence microscopy (46). In these assays, probes tagged with the Texas red fluorophore were used. After overnight hybridization with *Hspb1*-specific RNA probes at 37°C, the sections were washed with 2X SSC buffer and then blocked using BSA. Sections were then incubated with TDRD7 primary antibody (rabbit) (17) for 1 h at 37°C. Sections were blocked again and then incubated with anti-rabbit secondary antibody tagged with Cy5. The samples were washed with 1X PBS, stained with DAPI and mounted for imaging as described (47). While individual cells could not be discerned (as co-staining with phalloidin or WGA was not performed to minimize the number of experimental challenges/treatments), this analysis did provide a spatial context within the fiber compartment with regard to the association of TDRD7 protein with Hspb1 mRNA. Imaging was performed using a Nikon TiE inverted fluorescence microscope with 100× oil objective, equipped with a fully automated stage, a cooled CCD back-illuminated PIXIS 1024B camera and a MetaMorph (BioVision, Inc.) image acquisition software. The acquired images were analyzed and processed using a custom-written MATLAB program as described (48, 49).

### *X. tropicalis* breeding and injections

Wild-type *X. tropicalis* adults (male and female) were purchased from the NASCO. Methods involving live animals were carried out in accordance with the guidelines and regulations approved and enforced by the Institutional Animal Care and Use Committees at the University of Delaware. Morpholinos (MOs) were designed using Gene Tools, LLC, OR. The Hspb1 MO is a 25-mer with the sequence 5′ GTA TTC TGC GTT CTG ACA TTT TC 3′. The control MO is the standard control MO obtained from Gene Tools with the sequence 5′ CCT CTT ACC TCA GTT ACA ATT TAT A 3′. Embryos were collected and injected with a PLI-100A microinjector (Harvard Apparatus) as described previously (106). Control or HSPB1 morpholino (1.5 ng per blastomere) was injected into a single dorsal-animal blastomere at the eight-cell stage; Alexa Fluor 568 dextran (Invitrogen #D22912) was co-injected as a lineage tracer. For the rescue experiments, mouse Hspb1 mRNA was generated by *in vitro* transcription as described (107) and co-injected with the HSPB1 morpholino (50 pg mRNA per blastomere). Injected embryos were cultured in 0.1X MBS to desired stages, and eye phenotypes were observed using a Zeiss Axiozoom.v16 epifluorescence microscope.

### *X. tropicalis* phenotype scoring and statistics

For examining *X. tropicalis* eye phenotypes, injected embryos were allowed to develop to stage 42 and scored for eye defects. The percentages of normal and reduced phenotypes were calculated for injected embryos obtained from multiple independent experiments, and chi-squared tests were performed to compare the phenotypes in different treatment groups. Images of eyes (bright-field) were taken with a Zeiss Axiozoom.v16 epifluorescence microscope.

### Histology and DAPI staining in *X. tropicalis*

*X. tropicalis* embryonic tissue was fixed in 0.1 M sodium cacodylate buffer (SCB) (Electron Microscopy Sciences (EMS), #11653) containing 2% glutaraldehyde (EMS, #16020) and 2% paraformaldehyde (EMS, #15710) and stored at 4°C until further processing. On day 2, the tissue was washed three times (15 min each wash) in 0.1 M SCB and fixed for 2 h in 0.1 M SCB containing 1% osmium tetroxide (EMS, #19110). The tissue was washed in Nanopure water three times (15 min each wash) and dehydrated in acetone (EMS, #10016) series (25, 50, 75, 95%) for 15 min each and incubated in 95% acetone overnight at 4°C. On day 3, the tissue was dehydrated in 100% anhydrous acetone for two times (15 min each wash) and infiltrated for 15 min with 1:1100% acetone propylene oxide (EMS, #20411) followed by 100% propylene oxide for 15 min. Additionally, the tissue was infiltrated with EMBed-812 resin:propylene oxide (EMBed-812 resin-EMS, #14120) at the ratios of 1:3, 1:2, 1:1, 2:1 and 3:1 for 1 h each, respectively. The tissue was then infiltrated in 100% EMBed-812 resin for 1 h (two times separately) and stored in 100% EMBed-812 resin at room temperature overnight. On day 4, the tissue was infiltrated with fresh 100% EMBed-812 resin for 1 h. The tissue was then embedded in aluminum weigh dishes (EMS, #70048–01) and polymerized at 60°C for 24 h. The tissue was sectioned and stained with 1% toluidine blue (EMS, #22050) in 1% borax (Acme, 20 Mule Team Borax), and coverslips were mounted using HistoChoice mounting media (Sigma, #H2904). The stained tissue was imaged using a Zeiss Axiophot light microscope (Carl Zeiss Microscopy, Thornwood, NY). For DAPI staining, *Xenopus* embryonic tissue was embedded in tissue freezing media, OCT (Tissue Tek, Torrance, CA), and immediately placed at −80°C to freeze samples. Followed by this, frozen sections (coronal) were prepared at 10 μm thickness. Sections were fixed with chilled 1:1 methanol/acetone at −20°C for 20 min followed by DAPI staining (40,6-diamidine-2-phenylidole-dihydrochloride; 1:1000 dilution, Life Technologies, Carlsbad, CA; #D21490). Slides were then washed three times with 1X PBS, mounted and stored at −20°C until imaging using Zeiss LSM 880 confocal.

## Supplementary Material

Supplementary_Fig_S1_ddaa096

Supplementary_Fig_S2_ddaa096

Supplementary_Table_S1_ddaa096

Supplementary_Table_S2_ddaa096

Supplementary_Table_S3_ddaa096

Supplementary_Table_S4_ddaa096
