# Supplementary figures and images for: The Tudor-domain protein TDRD7, mutated in congenital cataract, controls the heat shock protein HSPB1 (HSP27) and lens fiber cell morphology
Source: Hum Mol Genet. 2020 May 18;29(12):2076–97. doi: 10.1093/hmg/ddaa096 (PMC7390939; doi:10.1093/hmg/ddaa096)

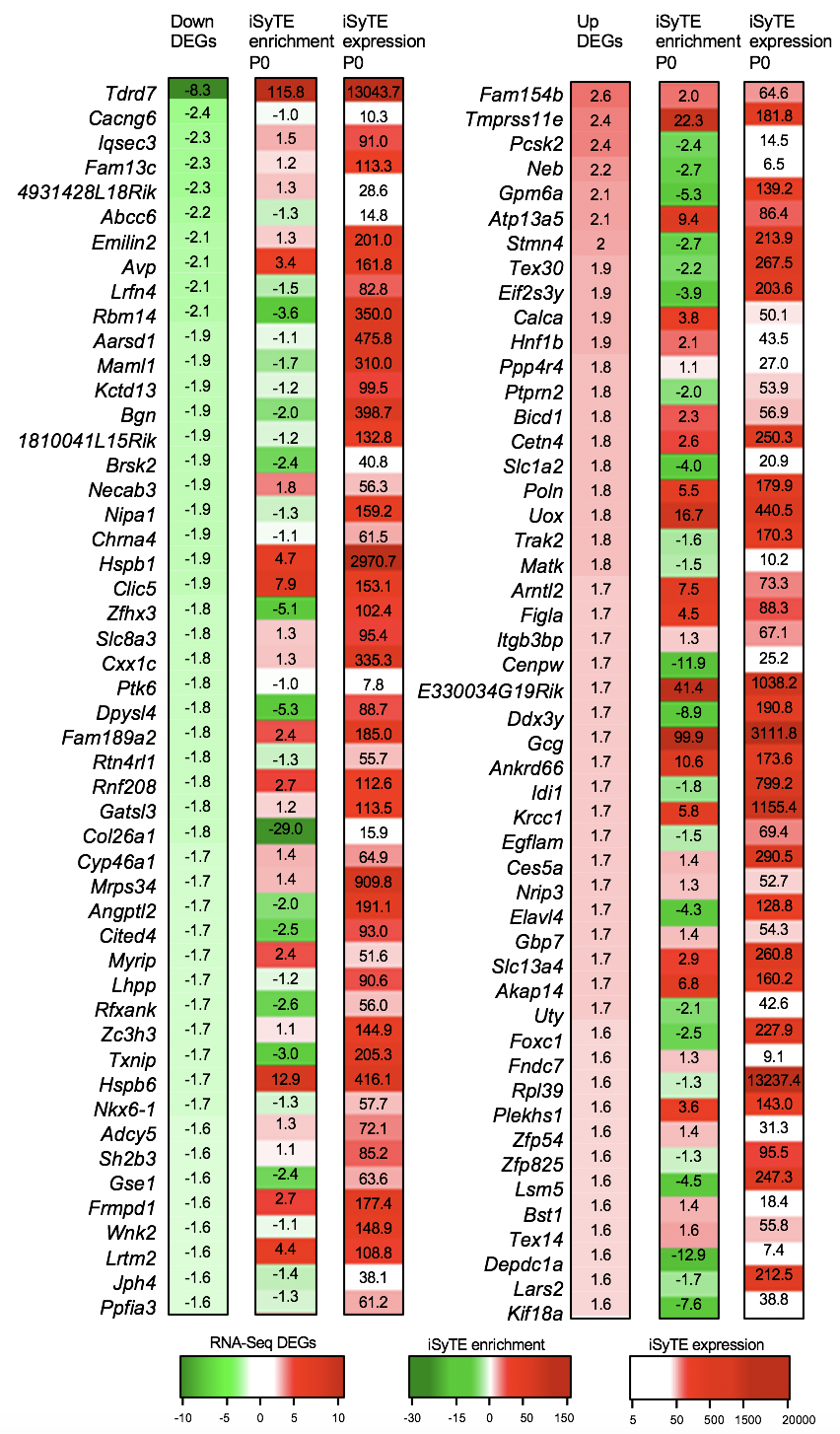

Supplement: Supplementary_Fig_S1_ddaa096 [file supplementary_fig_s1_ddaa096.png]

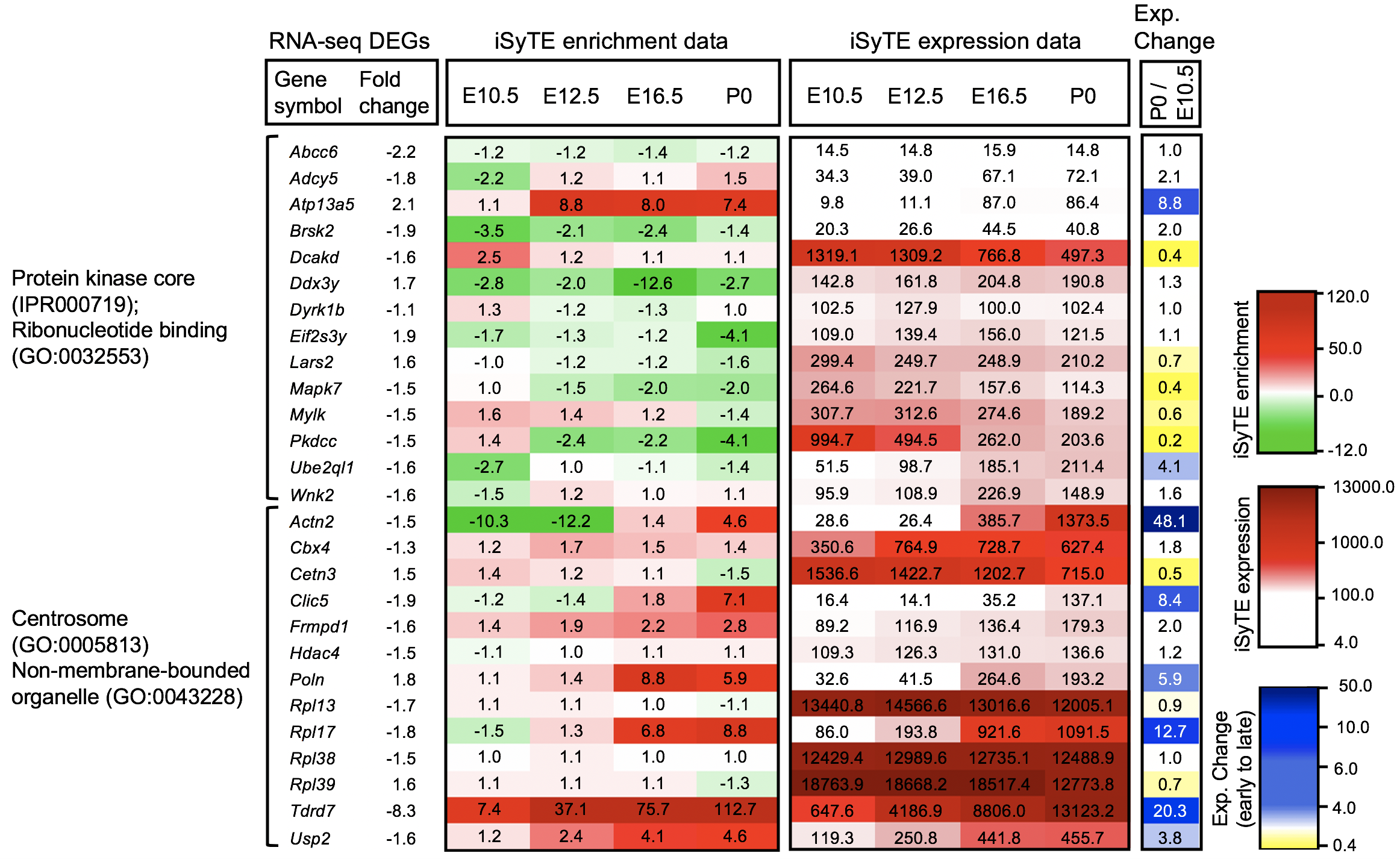

Supplement: Supplementary_Fig_S2_ddaa096 [file supplementary_fig_s2_ddaa096.png]
